# Appropriate Assignment of Fossil Calibration Information Minimizes the Difference between Phylogenetic and Pedigree Mutation Rates in Humans

**DOI:** 10.3390/life8040049

**Published:** 2018-10-22

**Authors:** Renata T. Capellão, Elisa M. Costa-Paiva, Carlos G. Schrago

**Affiliations:** Department of Genetics, Federal University of Rio de Janeiro, Rio de Janeiro 21941-902, Brazil; renatacapellao@gmail.com (R.T.C.); elisapolychaeta@gmail.com (E.M.C.-P.)

**Keywords:** species tree, primate evolution, speciation, coalescent, generation time

## Abstract

Studies that measured mutation rates in human populations using pedigrees have reported values that differ significantly from rates estimated from the phylogenetic comparison of humans and chimpanzees. Consequently, exchanges between mutation rate values across different timescales lead to conflicting divergence time estimates. It has been argued that this variation of mutation rate estimates across hominoid evolution is in part caused by incorrect assignment of calibration information to the mean coalescent time among loci, instead of the true genetic isolation (speciation) time between humans and chimpanzees. In this study, we investigated the feasibility of estimating the human pedigree mutation rate using phylogenetic data from the genomes of great apes. We found that, when calibration information was correctly assigned to the human–chimpanzee speciation time (and not to the coalescent time), estimates of phylogenetic mutation rates were statistically equivalent to the estimates previously reported using studies of human pedigrees. We conclude that, within the range of biologically realistic ancestral generation times, part of the difference between whole-genome phylogenetic and pedigree mutation rates is due to inappropriate assignment of fossil calibration information to the mean coalescent time instead of the speciation time. Although our results focus on the human–chimpanzee divergence, our findings are general, and relevant to the inference of the timescale of the tree of life.

## 1. Introduction

The study of mutation rates constitutes a fundamental problem in genetics and evolution [1,2]. Traditionally, the rate at which mutations occur has been measured on a generational basis [3,4]. However, with the advent of the molecular clock theory in the 1960s, mutation rates have also been measured per year [5]. In this sense, the age of the *Homo–Pan* split, gathered from the fossil record, has been widely used as calibration information for the estimation of the yearly substitution rates in several human loci [6], but only with the sequencing of the genomes of humans and chimpanzees, calculation of whole-genome substitution rates became feasible. It was shown that the genome-wide genetic divergence between humans and chimpanzees was roughly 0.012 substitutions per site [7,8,9]. Assuming that the age of the *Homo–Pan* split is approximately 7 million years ago (Ma) [6,10], a yearly substitution rate of 0.09 × 10^−8^ substitutions/nucleotide site/year (s/s/y) was inferred.

Apart from phylogenetic estimates relying on the *Homo–Pan* fossil calibration, the advent of high throughput sequencing technologies allowed the inference of the whole-genome mutation rate per nucleotide site by genome wide comparisons and direct estimates of de novo mutations of parent–offspring trios, with estimates varying from 0.96 to 1.2 × 10^−8^ substitutions/nucleotide site/generation (s/s/g) [11]. A similar estimate was calculated for Western chimpanzees (1.2 × 10^−8^ s/s/g) based on whole-genome comparison in pedigrees [12]. To convert pedigree-based generational rates into per-year rates, the duration (in years) of the generation time is required. On that account, to make pedigree and phylogenetic mutation rates comparable, the generation time of the ancestor of humans and chimpanzees should be set at ~12 years. However, this value is too small when compared to previous studies that calculated ancestral *Homo–Pan* generation time ranging from 15 to 26 years [8,13,14]. Consequently, genome-wide phylogenetic mutation rates estimated using the *Homo–Pan* fossil calibration are higher than pedigree-based estimates. Under strict neutrality, however, both values are expected to be equivalent [1], and exchanges of phylogenetic and pedigree rates evidently impact the reconstruction of the hominine evolutionary timescale [15].

**Figure 1:**
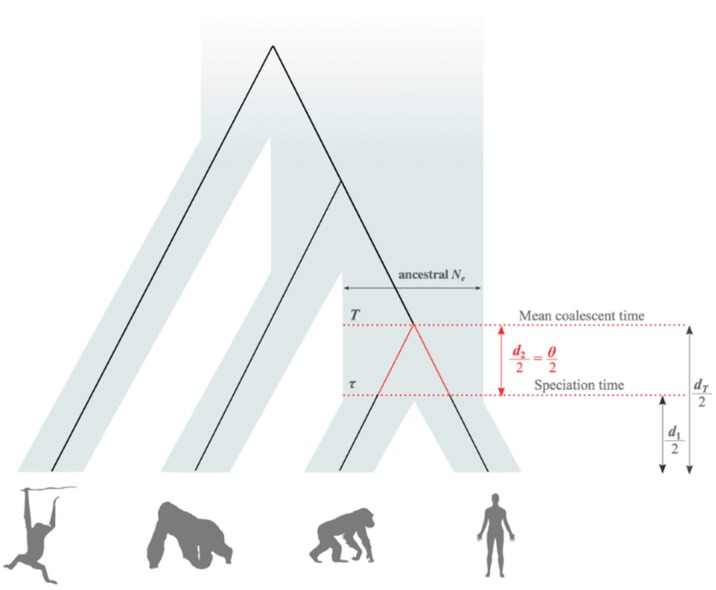
Difference between the genetic divergence (T), which is calculated by the mean coalescent time between human–chimpanzee gene pairs (*d*_T_/2), and the speciation time (τ), which is the time of the genetic isolation between species (*d*_1_/2). *d*_T_ is the total genetic distance between humans and chimpanzees; *d*_1_ is the total genetic distance accumulated after the speciation time τ; and *d*_2_ is the amount of sequence divergence existent in the ancestral population, before the complete genetic isolation between species. To correctly calibrate the hominoid timescale, the age of the oldest fossil belonging to the *Homo* or *Pan* lineages should be assigned to τ, which is equivalent to the time that *d*_1_/2 requires to accumulate. Assigning the calibration to T will overestimate the phylogenetic mutation rate, because *d*_T_ > *d*_1_. The values of *d*_T_, *d*_1_, and *d*_2_ are measured in substitutions/site.

Previous works have proposed that the discrepancy between phylogenetic and pedigree mutation rates can be explained by a general reduction in the mutation rate per year during the evolution of great apes, due to gradual increase in generation times towards the lineage that gave rise to modern humans [16,17]. This reduction, known as the hominoid slowdown hypothesis, was initially reported based on a limited amount of genetic data [18,19], but was later confirmed with genome-wide datasets [20,21]. Nonetheless, factors other than generation time might account for the discrepancy between pedigree and phylogenetic rates, without requiring the slowdown hypothesis. Misspecification of fossil calibration information is the most obvious example [22]. This is because phylogenetic rates are estimated using calibration information from the paleontological record, which brings a number of limitations. Besides the scarcity of fossil findings, the ages of fossils rarely (if ever) correspond to the time of the average genetic divergence across loci between species, i.e., the mean genome-wide coalescent time [23]. At best, fossils register the minimum age of the speciation time. In classical molecular phylogenetics, however, it is customary to consider the average genetic divergence between species (*d*_T_) as equal to the total amount of genetic divergence (*d*_1_) accumulated after the age of the complete isolation between species (τ) (Figure 1). We expect, however, the difference between *d*_T_ and *d*_1_ to be significant in cases such as the comparison of recently isolated species or when the effective population size of the ancestral lineage was large [24]. Thus, the assignment of fossil calibration information to T instead of τ overestimates the substitution rate [25,26]. In fact, this issue is unlikely to be ameliorated even if all the fossils of a lineage were perfectly recorded, because of discrepancies between the rates of phenotypic and genome evolution.

Using whole genomes, we were prompted to investigate whether the correct assignment of the *Homo–Pan* fossil calibration information to the speciation time τ instead of the mean coalescent time *T* leads to an accurate estimate of the phylogenetic rate of great apes, consequently reducing the discrepancy between the phylogenetic rate and the empirically measured human pedigree rates using a between-species phylogenetic data of great apes. To do so, we assembled a dataset consisting of orthologous genomic regions randomly collected across the genomes of the great apes.

## 2. Materials and Methods

In this study, we investigated whether the incorrect use of *Homo–Pan* calibration information, as a result of incorrectly accounting for the amount of polymorphism present prior to the speciation of humans and chimpanzees (*d*_2_ = *θ*, Figure 1), leads to an overestimation of the phylogenetic mutation rate. Therefore, we expect that, if the calibration is correctly assigned to the speciation time τ (*d*_1_/2) instead of *T* (*d*_T_/2), the estimated phylogenetic mutation rate will decrease and approach the short-term pedigree rates reported in the literature from empirical analyses (Table 1).

### 2.1. Sequences and Alignments

We composed a phylogenetic dataset consisting of 15,744 alignments of 5000 bp segments interspaced by more than 10^7^ bp along the genome that were collected randomly across the syntenic alignments of *Homo*, *Pan*, *Gorilla*, and *Pongo* available in Ensembl’s Compara database (www.ensembl.org/info/genome/compara). *Pongo* was used as outgroup throughout the analyses. To avoid methodological biases associated with rate heterogeneity among lineages, all segments analyzed failed to reject the molecular clock at 5% significance level by employing the likelihood ratio test (LRT) as implemented in the baseml program of the PAML 4 package [39].

### 2.2. Average Coalescent Times and Speciation Times

The total genetic distance between any pair of nucleotide sequences collected from different species is composed of two processes (Figure 1): (1) the average number of substitutions per site accumulated after the speciation process (*d*_1_); and (2) the average number of substitutions per site accumulated before the speciation, in the ancestral population that gave rise to both species being compared (*d*_2_ = *θ*). If the speciation time of the species pair is τ, Part (1) is given by *d*_1_ = 2µτ, whereas Part (2) is given by the expectation of coalescent theory. If the effective size of the ancestral population was *N*_e_, the expected waiting time of a pair of alleles is exponentially distributed with λ = 1/2*N_e_*, i.e., with mean = 2*N_e_*. Then, the average number of substitutions between a pair of alleles in the ancestral population is given by *d*_2_ = 2µ (2*N_e_*), which equals 4*N_e_*µ. This expression describes the fundamental mutation parameter *θ*. Therefore, the total genetic distance between a pair of species is given by *d*_T_ = *d*_1_ + *θ*. Evidently, if the molecular clock holds, i.e., mutation rates were homogeneous across branches, the age of the average coalescent time, as measured in substitutions per site (s/s), is *d*_T_/2. Likewise, the age of the speciation time (in s/s) is *d*_1_/2 (Figure 1).

The number of substitutions per site accumulated since the mean coalescent time of humans and chimpanzees, *d*_T_/2, was estimated by maximum likelihood in PhyML 3 [40]. The collected genomic segments were concatenated into a supermatrix of 78,720,000 bp. The model of sequence evolution employed to account for multiple hits was chosen by in HyPhy employing the LRT implemented in modeltest (GTR + G + I). By comparing the whole genomes of humans and chimpanzees, previous works have calculated *d*_T_/2 to be 0.0062 s/s [7]. Therefore, we used this value as a gold standard to evaluate the estimates obtained with our datasets.

### 2.3. Estimation of Mean Speciation Times τ

Speciation time between humans and chimpanzees, i.e., *d*_1_/2 (Figure 1), was inferred with the BPP software [41]. This software performs Bayesian inference of the speciation time and ancestral population sizes via a Markov chain Monte Carlo (MCMC) algorithm using the method of Rannala and Yang [14]. We ran BPP’s MCMC analysis independently twice. In each run, chains were sampled every 1000th generation until 20,000 samples were obtained. Because BPP runs a Bayesian algorithm, prior distributions for parameters must be assigned. In this regard, a gamma distribution G (α = 2, β = 200) was used as prior for the *θ* parameter. This is the default distribution used in BPP, and it contemplates a wide range of *θ* values reported in empirical datasets. For the τ parameter at the root node, which is the speciation time between humans and orangutans measured in substitutions/site, we assigned a gamma prior G (α = 16.7, β = 1264.0), which has a mean equal to half the genetic distance between humans and orangutans (0.013 s/s). This prior was obtained by fitting a gamma density to the distribution of *d*_T_/2 between humans and orangutans estimated from the loci analyzed. The MASS package of the R programming environment was used to fit the gamma distribution.

### 2.4. Fossil African Great Apes and Humans

To estimate absolute yearly mutation rates (substitutions/site/year) from speciation times, one must divide the estimate of τ obtained in BPP, which is measured in s/s (*d*_1_/2), by the absolute speciation time between *Homo* and *Pan*, measured in years. Although the fossil record of the great apes is not complete, it is generally regarded that *Sahelanthropus tchadensis* from Chad is one of the earliest species belonging to the evolutionary lineage leading to humans after the genetic isolation (speciation) from the chimpanzee lineage [10]. Stratigraphic analyses placed this fossil at approximately seven million years ago (Ma). In this study, we thus considered this age as the speciation time between *Homo* and *Pan*. We reaffirm that we assigned this calibration to *d*_1_/2 instead of *d*_T_/2 to measure the yearly phylogenetic rate. To account for the uncertainty associated with the fossil calibration information assigned to the age of the *Homo–Pan* speciation, we also calculated the phylogenetic rate using a wide range of paleontological speciation times, from 3.5 to 15 Ma.

### 2.5. Generation Times

To obtain the estimates of evolutionary rates per generation, the time duration (in years) of a single generation of the ancestor of humans and chimpanzees is needed. In the literature, the value of this parameter often ranges from 15 to 20 years [8,42,43]. Schrago [13], using the estimates for living species reported by Langergraber et al. [44], performed ancestral state reconstruction of continuous characters to infer the ancestral generation time at 26.3 years. An estimate close to 26 years was also corroborated by the applying other analytical approaches [45]. Because this issue is still contentious, we calculated the phylogenetic rate setting the ancestral *Homo–Pan* generation time to 15, 20 and 26.3 years. To investigate the impact of this value on phylogenetic rates, we further examined a wide range of generation times, varying from 10 to 30 years.

## 3. Results and Discussion

The average number of substitutions per site accumulated since the mean coalescent time (*d*_T_/2) was 0.00625 s/s, which was close to the estimate from human–chimpanzee whole-genome comparison (Table 2). However, when the difference between mean coalescent and speciation times was accounted for, the average number of substitutions accumulated since the complete genetic isolation of humans and chimpanzees, the speciation time τ (*d*_1_/2), decreased to 0.00473 s/s. Therefore, considering 7 Ma as the absolute age of the speciation of both species, the yearly evolutionary rate was inferred at 0.068 × 10^−8^ s/s/y.

If the generation time of the ancestor of humans and chimpanzees was 15 years, the generational mutation rate was inferred at approximately 0.99 × 10^−8^ s/s/g, when this value was increased to 26.3 years, the generational mutation rate shifted to approximately 1.77 × 10^−8^ s/s/g (Table 3). Thus, when fossil calibration was correctly placed, the generational mutation rates calculated with the phylogenetic dataset were closer to the empirical estimates obtained from short-term pedigree-based analyses of humans reported in Table 1.

**Figure 2:**
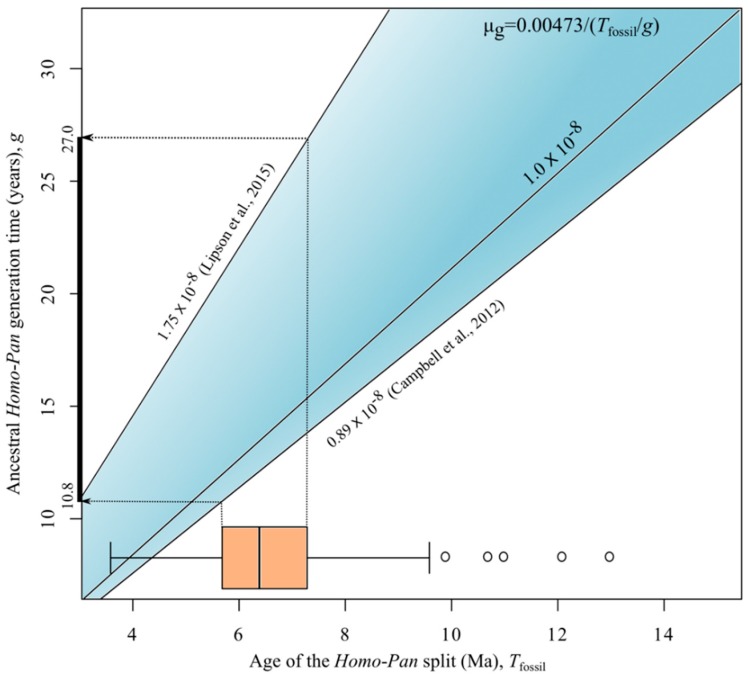
Plot of the estimated pedigree rates (µ_g_) assigning the calibration information to 0.00473 s/s, i.e., the inferred average number of substitutions per site accumulated since the speciation of *Homo* and *Pan*. To calculate the pedigree rates, a wide range of ancestral *Homo–Pan* generation times (g) and ages of the human–chimpanzee speciation (*T*_fossil_) were used. The blue area establishes the minimum and maximum limits of µ_g_ obtained from empirical the studies of Campbell et al. [30] (lower estimate) and Lipson et al. [34] (upper estimate), respectively. We also show the line for the average rate calculated from the estimates published so far (~1.0 × 10^−8^ s/s/g). The boxplot shows the distribution of estimates of *T*_fossil_ from various studies available in the timetree.org database.

When varying both the values of the fossil calibration age and the ancestral generation time, the mutation rates calculated using our estimated *Homo–Pan* speciation time (*d*_1_/2 = 0.00473 s/s) were within the range of empirical pedigree rates, which varies from 0.89 to 1.75 s/s/g, corresponding to the minimum and maximum values reported in Campbell et al. [30] and Lipson et al. [34] (Figure 2). For instance, if the *Homo–Pan* split took place between 5.7 and 7.3 Ma, which is the range that encloses most estimates reported in the literature (timetree.org) [46], to account for the minimum and maximum values of the pedigree mutation rate, the generation time of the *Homo–Pan* ancestor should be as low as 10.8 and as high as 27.0 years (Figure 2). Within this range, phylogenetic and pedigree mutation rates are statistically equivalent.

The magnitude of the difference between the phylogenetic rate estimates and the short-term pedigree rates was thus conditional on both the values of the generation time of the ancestor of humans and chimpanzees and the age of the *Homo–Pan* split. Because the uncertainty associated with the ancestral *Homo–Pan* generation time is arguably more contentious than the speciation time of both lineages, we focused our analysis on this variable, setting the split at 7 Ma. With g = 15 years, estimates of phylogenetic rates were indistinguishable from the values inferred by previous whole genome pedigree studies (Table 1). When generational time increased, the estimates also increased, departing from pedigree-based estimates. Kong et al. [31] showed that the number of human de novo mutations increases with the paternal age in order of about two mutations per year, while this parameter is not significantly affected by the maternal age at the time of conception. These results corroborate the idea that a larger number of divisions during spermatogenesis is responsible for the increase of the generational mutation rate [47]. The long-term, phylogenetic, effects of these results are yet to be explored.

Langergraber et al. [44] inferred a generation time of 19 years for gorillas and 25 years for chimpanzees from genetic parentage data from a large number of individuals from both species. Assuming a short-term rate between 0.97 × 10^−8^ and 1.36 × 10^−8^ s/s/g, the authors suggested that mutation rate for humans and African apes lies between 0.03 × 10^−8^ and 0.07 × 10^−8^ s/s/y. However, the maximum value of the pedigree generational estimates adopted in that study corresponds to a rate estimated using highly polymorphic microsatellites (1.36 × 10^−8^ s/s/g) which are expected to have a higher rate compared to the whole genome estimates [48]. If estimates from fast evolving microsatellites are ruled out and only whole genome pedigree generational rates are considered, we found that the rate estimated here lies within the 95% confidence interval of previous pedigree-based studies. On the other hand, Lipson et al. [34] reported a high genome-wide rate of 1.65 ± 0.10 × 10^−8^ mutations per generation using an approach based on the relationship between local heterozygosity in diploid genomes, recombination rates, and genetic distance. The rate estimated by Lipson and colleagues approaches the phylogenetic rate estimated here assuming an ancestral generation time between 20 and 26.3 years. Differently from pedigree comparisons, Lipson et al.’s new calibration method, dubbed as the ancestral recombination density, avoids direct individual genome comparisons and therefore is free from the bias related to the distinction between de novo mutations and sequencing errors in pedigree-based analysis, which are expected in direct comparisons of single generation data of a small subset of individuals [11].

Because the phylogenetic rate should not be higher than the pedigree evolutionary rate at neutral loci, we argue that the generation time of the ancestor of *Homo* and *Pan* was likely lower than 26.3 years, lying between 15 and 20 years. It is worth mentioning that the credibility interval of previously reported estimates of *Homo*/*Pan* ancestral generation time included this 15–20-year range [13].

The inference of the *Homo–Pan* speciation time τ assumed a simple speciation model of no gene flow taking place after the speciation process, and recent works have reported post-speciation gene flow in primates [49,50]. Although models that allow for post-speciation gene flow were proposed [51,52], we have opted for investigating a simpler scenario in our study to avoid overparameterization. In fact, gene flow will reduce the average genetic distance between species (coalescent time), decreasing the phylogenetic mutation rate estimate, and minimizing the discrepancy between phylogenetic and pedigree rates.

Although our study focused on the divergence between humans and chimpanzees, overestimation of the phylogenetic rate by incorrect assignment of the calibration information to the coalescent time instead of the speciation time affects any divergence in the tree of life. Because the difference between the coalescent time and the speciation time depends on the effective population size of the ancestor of the two daughter lineages, the larger the ancestral *N*_e_, the larger will be the overestimation of the phylogenetic rate. Indeed, very large *N*_e_s were reported for lineages from plants (2,290,000, [53]) to invertebrates (72,584,531, [54]) [55]. In this sense, studies that borrow evolutionary rates from closely related lineages to infer dated phylogenies should be cautious of such bias. Using overestimated rates will lead to younger divergence times along the tree of life.

## 4. Conclusions

We showed that, using a long-term (phylogenetic) dataset of African great apes, it is feasible to estimate a phylogenetic rate that is statistically comparable to the estimates of short-term (pedigree) human mutation rates. Using a dataset of randomly sampled orthologous genomic segments under the molecular clock, phylogenetic estimates approached pedigree-based rate estimates when the fossil calibration was correctly assigned to the *Homo–Pan* speciation time, instead of the mean genetic divergence time. The long-term/short-term agreement, however, required that the generation time of the *Homo*–*Pan* ancestor lie between 15 and 20 years. We argue, therefore, that estimates of human mutation rates behaved as theoretically predicted and previous incongruences were partly caused by incorrect handling of fossil calibration information. Although we analyzed an example from primates, incorrect assignment of calibration to coalescent times should impact any divergence in the tree of life, leading to overestimates of phylogenetic rates that would compromise the accurate timing of evolutionary divergences.

## Figures and Tables

**Table 1:** Estimates of human mutation rates per generation from several whole genome population-level studies. * Obtained through the comparison of contemporaneous and ancient human sequences and converted from a yearly estimate of 0.38–0.49 × 10^−8^, assuming a human generation time of 29 years.

Study	Mean Rate (s/s/g)
1000 Genomes Project Consortium [27]	1.0–1.2 × 10^−8^
Roach et al. [28]	1.1 × 10^−8^
Conrad et al. [29]	0.97–1.17 × 10^−8^
Cambpell et al. [30]	0.89–1.43 × 10^−8^
Kong et al. [31]	1.20 × 10^−8^
Michaelson et al. [32]	1.0 × 10^−8^
Fu et al. [33] *	1.10–1.42 × 10^−8^
Lipson et al. [34]	1.55–1.75 × 10^−8^
Besenbacher et al. [35]	1.16–1.38 × 10^−8^
Rahbari et al. [36]	1.13–1.43 × 10^−8^
Amster and Seela [37]	1.2 × 10^−8^
Wong et al. [38]	1.05 × 10^−8^

**Table 2:** Estimates of the mean *Homo/Pan* genetic distances and absolute yearly evolutionary rates by applying the paleontological calibration at the mean coalescent time (*T*) and the speciation time (τ).

Mean Coalescent Time (*T*)		Speciation Time (τ)	
Genetic Distance (*d*_T_/2) ^1^	Yearly Evolutionary Rate ^2^	Genetic Distance (*d*_1_/2) ^1^	Yearly Evolutionary Rate ^2^
0.00625 *	0.089 × 10^−8^	0.00473 ± 0.000040	0.067–0.068 × 10^−8^

^1^ In substitutions/site; ^2^ in substitutions/site/year, adopting the age of the *Homo*/*Pan* split at 7 Ma; * the errors associated with the maximum likelihood estimates approached zero, because the number of sites analyzed was very large.

**Table 3:** Estimates of generational evolutionary rates (substitutions/nucleotide site/generation) using phylogenetic information from the inferred speciation time, assuming ancestral *Homo–Pan* generation times of 15, 20 and 26.3 years.

Ancestral *Homo–Pan* Generation Time (Years)	Evolutionary Rate
15	0.99 × 10^−8^–1.03 × 10^−8^
20	1.33 × 10^−8^–1.37 × 10^−8^
26.3	1.75 × 10^−8^–1.81 × 10^−8^
